# Tumor Necrosis Factor Superfamily 14: A Potential Predictor of Atrial Fibrillation Recurrence After Catheter Ablation

**DOI:** 10.1002/clc.70283

**Published:** 2026-04-20

**Authors:** Dongtao Zhou, Fang Liu, Tong Liu, Mengmeng Li, Chenxi Jiang, Ribo Tang, Wei Wang, Xin Zhao, Changyi Li, Changqi Jia, Man Ning, Li Feng, Dan Wen, Jing Lin, Hui Zhu, Yuexin Jiang, Xueyuan Guo, Songnan Li, Chao Jiang, Ning Zhou, Caihua Sang, Deyong Long, Xin Du, Jianzeng Dong, Changsheng Ma

**Affiliations:** ^1^ Department of Cardiology, Beijing Anzhen Hospital, National Clinical Research for Cardiovascular Diseases Capital Medical University Beijing China

**Keywords:** ATLAS score, atrial fibrillation recurrence, catheter ablation, predictive value, tumor necrosis factor superfamily 14

## Abstract

**Background:**

Tumor necrosis factor superfamily 14 (TNFSF14) has been implicated in the pathogenesis of cardiovascular disease, including atrial fibrillation (AF). However, its role in predicting AF recurrence after catheter ablation (CA) remains unexplored.

**Objective:**

This study aimed to evaluate the predictive value of TNFSF14 for AF recurrence post‐ablation.

**Methods:**

A total of 263 AF patients undergoing CA were enrolled and stratified into two groups based on AF recurrence during a mean follow‐up of 363 ± 144 days. Plasma TNFSF14 levels were measured by enzyme‐linked immunosorbent assay (ELISA). Cox proportional hazards models were employed to examine the association between TNFSF14 levels and AF recurrence, while Receiver Operating Characteristic (ROC) analysis was used to assess predictive performance.

**Results:**

AF recurrence occurred in 81 patients (30.8%). Patients with recurrence exhibited significantly higher baseline TNFSF14 levels (1.21 ± 0.24 vs. 1.02 ± 0.29 ng/mL, *p* < 0.001). Elevated TNFSF14 levels were independently associated with AF recurrence (Adjusted hazard ratio (aHR): 3.65, 95% CI: 2.19–6.09, *p* < 0.001). ROC analysis demonstrated moderate predictive power for TNFSF14 (AUC: 0.70). Incorporating TNFSF14 levels into the ATLAS score and BNP significantly enhanced the predictive performance for recurrence, as evidenced by improved time‐dependent AUC, decision curve analysis, net reclassification improvement (NRI: 0.36, *p* < 0.001) and integrated discrimination improvement (IDI: 0.08, *p* = 0.012).

**Conclusions:**

TNFSF14 is a promising biomarker for predicting AF recurrence after CA. It holds potential for inclusion in future personalized risk models for AF recurrence.

## Graphical abstract

TNFSF14 levels may reflect atrial fibrosis and inflammation. High TNFSF14 level was associated with increased atrial fibrillation recurrence after catheter ablation. Incorporating TNFSF14 into conventional risk models can improve predictive performance and aid individualized post‐ablation management.

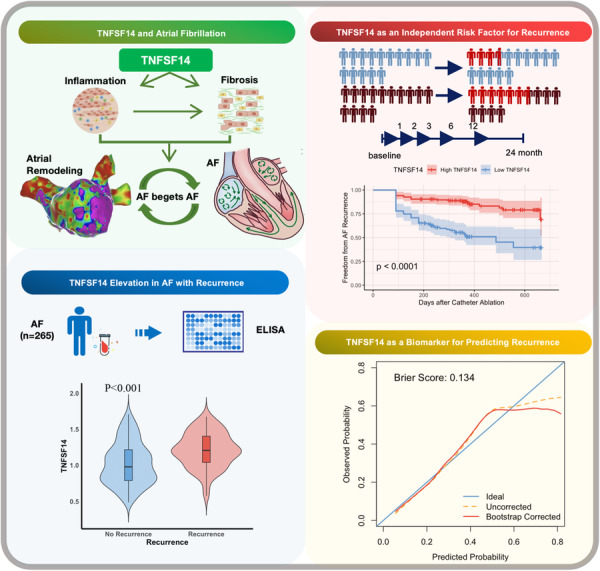

**Abbreviations**
AF: atrial fibrillation
BNP: B‐type natriuretic peptide
CA: catheter ablation
CRP: C‐reactive protein
ECG: electrocardiograph
HVEM: herpesvirus entry mediator
LAVI: Left atrial volume index
LTβR: lymphotoxin β receptor
TNFSF14: tumor necrosis factor superfamily 14

## 1 Introduction

Atrial fibrillation (AF) and its recurrence following catheter ablation pose significant challenges in clinical management. The recurrence of AF can substantially impair patients’ quality of life, elevate their risk of stroke, and contribute to long‐term morbidity [1]. Furthermore, recurrent AF is closely associated with an increased risk of heart failure [2], diminished exercise capacity, and heightened psychological distress, including anxiety and frustration [3]. Despite substantial advancements in catheter ablation techniques, the recurrence rate remains notably high, and the underlying mechanisms are not yet fully elucidated. Atrial remodeling, characterized by structural and functional alterations in atrial tissue, has been identified as a critical factor in the pathogenesis of arrhythmogenesis, which is now considered central to the initiation, maintenance, and recurrence of AF [4, 5]. The identification of reliable biomarkers for predicting AF recurrence is therefore essential to improve patient outcomes and guide the development of personalized therapeutic strategies.

Tumor Necrosis Factor Superfamily 14 (TNFSF14), also known as LIGHT or CD258, is a member of the tumor necrosis factor superfamily and plays a key role in regulating the T cell immune response [6]. Moreover, T cell immune responses contribute to both inflammation and fibrosis, which are closely associated with atrial fibrillation [7, 8]. Besides, previous studies have demonstrated elevated TNFSF14 levels in AF patients compared to healthy controls, and its role in cardiac fibrosis has been suggested through macrophage M2 polarization via the PI3K‐γ/SGK1 pathway [9]. However, the relationship between TNFSF14 and AF recurrence following CA remains unexplored. This study aimed to investigate whether TNFSF14 levels could serve as a biomarker for predicting AF recurrence after ablation.

## 2 Methods

### 2.1 Study Population

**Figure 1:**
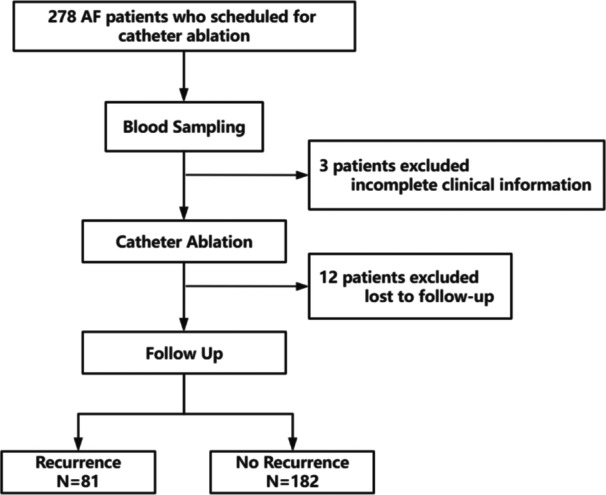
Flow diagram of inclusion and exclusion of study subjects. AF = atrial fibrillation.

This prospective cohort study enrolled 278 symptomatic non‐valvular AF patients scheduled for catheter ablation at Beijing Anzhen Hospital between October 2022 and September 2023. The exclusion criteria included: systemic inflammatory disease, severe chronic obstructive pulmonary disease, acute coronary syndrome, malignancy, active infection within the last 2 weeks, severe aortic or mitral valve disease and pregnancy. All basic information was collected from the medical record system of Anzhen Hospital and biochemical parameters were assessed at the central laboratory of Beijing Anzhen Hospital. The study flowchart was presented in Figure 1. Ethical approval was obtained from the Ethics Committees of Beijing Anzhen Hospital, Capital Medical University, China (KS2023082). Written informed consent was obtained from all participants, and the study was conducted in accordance with the principles of the Declaration of Helsinki.

### 2.2 Laboratory Measurements and TNFSF14 Grouping

Venous blood samples were collected prior to ablation. The concentrations of TNFSF14 were quantified using enzyme‐linked immunosorbent assay (ELISA). Plasma was separated via centrifugation at 3000 g for 15 min. TNFSF14 levels were measured using a commercially available ELISA kit (Human TNFSF14 ELISA Kit, YX‐201406H, both intra‐assay and inter‐assay variability< 15%), following the manufacturer's instructions. Standard curves were generated using serial dilutions of recombinant human TNFSF14, and plasma concentrations were determined by extrapolation using a log–log linear regression model (Supplemental Methods, Samples and analyses).

### 2.3 Catheter Ablation

All ablation procedures were performed using radiofrequency energy and guided by a three‐dimensional electroanatomic mapping system (CARTO3; Biosense‐Webster, Inc., Diamond Bar, CA). For patients undergoing primary ablation, circumferential pulmonary vein isolation (CPVI) was routinely performed. In patients with persistent atrial fibrillation, three additional linear ablations—at the left atrial roof, the mitral isthmus, and the cavo‐tricuspid isthmus—were conducted. Additional interventions, such as vein of Marshall ethanol infusion and ablation of complex fractionated atrial electrograms, were performed at the operators’ discretion. The procedural endpoint was the achievement of pulmonary vein isolation and bidirectional conduction block of the additional linear lesion. In patients with prior ablation, electrophysiological examinations were conducted to evaluate these endpoints, and touch‐up ablation was performed as needed. Antiarrhythmic drugs (AADs) were usually continued for 3 months post‐ablation and systematically discontinued thereafter.

### 2.4 Follow‐Up and Outcome

Routine ECG or 24‐h Holter monitoring was performed for all patients at 1, 2, 3, 6, and 12 months post‐ablation. Additionally, if patients experienced symptoms suggestive of AF recurrence, immediate ECG or 24‐h Holter monitoring was conducted. Follow‐up assessments were conducted through outpatient visits or telephone interviews at 1, 2, 3, 6, and 12 months after ablation. AF recurrence was defined as the occurrence of atrial tachyarrhythmia lasting more than 30 s beyond a 3‐month blanking period.

### 2.5 Statistical Analyses

Continuous variables were expressed as mean ± standard deviation or median (interquartile range), while categorical variables were expressed as numbers (percentages). Differences in the baseline data between groups were analyzed using Student's *t*‐test, Mann‐Whitney U test and Pearson's chi‐square. The correlations between these variables were evaluated using the Spearman rank correlation coefficient.

The cut‐off value of TNFSF14 was determined based on the median level, and patients was categorized into high‐ or low‐ level groups to assess its association with AF recurrence.

Univariate and multivariate Cox regression analyses were conducted to determine independent risk factors for AF recurrence after ablation. Variables with values of *p* < 0.05 in the univariate analysis were included in the multivariate analysis. Receiver operating characteristic (ROC) curve analysis was conducted to determine the area under the curve (AUC), sensitivity, and specificity. The Kaplan–Meier method and log‐rank test were applied to compare the recurrence risk between different groups. Sensitivity analyses were performed by stratifying TNFSF14 levels using different grouping methods, including the mean value, cut‐off value of ROC, tertiles, and quartiles. Cox regression, analysis of differences between groups, and K–M survival analyses were repeated for each grouping approach.

Based on the results of multivariate regression analysis, a new model was constructed by incorporating the levels of TNFSF14 and B‐type natriuretic peptide (BNP) into the ATLAS score [10] (Supplemental Methods, ATLAS score) to improve the predictive ability for AF recurrence. Decision curve analysis was also employed to assess the comprehensive clinical utility across various threshold probabilities. Net reclassification improvement (NRI) and integrated discrimination improvement (IDI) metrics were calculated to determine whether the new model provided incremental predictive value beyond the ATLAS score.

R software (version 4.4.1) was utilized for conducting the statistical analysis. A two‐tailed *p*‐values < 0.05 was considered statistically significant for all comparisons.

## 3 Results

### 3.1 TNFSF14 Was Significantly Elevated in Patients With Recurrence During Follow‐Up

From October 2022 to September 2023, we enrolled 278 patients with AF who underwent CA at Beijing Anzhen Hospital in the study. We excluded 15 patients: three who refused to provide blood samples and 12 who were lost to follow‐up (Figure 1). Of the 263 patients with AF (mean age 62.6 ± 11.0 years, 61.6% male) included in our analyses, 81 (30.8%) experienced AF recurrence after ablation. Among the 263 patients, 192 (73.0%) received AADs during the blanking period, while 97 (36.9%) continued or initiated AADs beyond the blanking period due to recurrence, premature atrial complexes or atrial tachycardia episodes (< 30 s). The clinical, echocardiography and biochemical characteristics of the study population, stratified by the presence of recurrence, are summarized in Table 1.

**Table 1:** Baseline characteristics.

	Patients with recurrence (*N* = 81)	Patients without recurrence (*N* = 182)	*P*
Age, years	62.7 ± 11.1	62.6 ± 11.0	0.937
Woman	33 (40.7%)	68 (37.4%)	0.603
Height, cm	167 ± 9.84	168 ± 8.72	0.931
Weight, kg	74.7 ± 14.5	74.0 ± 12.0	0.658
BMI, kg/m^2^	26.5 ± 3.64	26.3 ± 3.26	0.660
HR, beats/min	74.0 (70.0,78.5)	75.0 (70.0,80.0)	0.116
SBP, mmHg	129 ± 15.5	128 ± 17.1	0.939
DBP, mmHg	79.0 ± 9.91	79.7 ± 12.3	0.650
Persistent AF	48 (59.3%)	75 (41.2%)	0.007*
AF Duration, year	3.00 (0.50,8.50)	2.00 (0.50,5.00)	0.020*
CHA2DS2‐VASc	2.00 (1.00,3.00)	2.00 (1.00,3.00)	0.314
HAS‐BLED	1.00 (0.00,1.00)	0.00 (0.00,1.00)	0.097
ATLAS score	7.90 ± 3.20	6.01 ± 3.35	< 0.001**
Current smoking	10 (12.4%)	25 (13.7%)	0.759
Current alcohol drinking	17 (21.0%)	29 (15.9%)	0.319
Redo	18 (22.2%)	31 (17.0%)	0.318
Heart failure	21 (25.9%)	37 (20.3%)	0.312
Ischemic stroke	11 (13.6%)	15 (8.24%)	0.181
Hypertension	45 (55.6%)	102 (56.0%)	0.941
CAD	16 (19.8%)	41 (22.5%)	0.614
Diabetes	14 (17.3%)	30 (16.5%)	0.872
Hyperlipidemia	24 (29.6%)	46 (25.3%)	0.461
Thyroid gland disease	7 (8.64%)	9 (4.95%)	0.247
LAVI, mL/m^2^	35.2 ± 15.8	25.4 ± 10.5	< 0.001**
LVEF, %	61.0 (58.0,65.0)	63.0 (58.0,66.0)	0.100
LVEDD, mm	48.0 (46.0,52.0)	46.5 (44.0,50.0)	0.001*
AAD_pre	48 (59.3%)	76 (41.8%)	0.009*
β blocker	20 (24.7%)	38 (20.9%)	0.491
CCB	16 (19.8%)	40 (22.0%)	0.684
ACEI/ARB	34 (42.0%)	55 (30.2%)	0.063
AAD_blank	62 (76.5%)	130 (71.4%)	0.388
AAD_post	36 (44.4%)	61 (33.5%)	0.090
TNFSF14, ng/mL	1.21 ± 0.24	1.02 ± 0.29	< 0.001**
BNP, pg/mL	234 ± 137	164 ± 129	< 0.001**
hsCRP, mg/L	1.17 (0.61,2.94)	0.94 (0.44,2.11)	0.041*
NLR	2.13 (1.57,3.03)	2.14 (1.55,2.73)	0.487
LDL, mmol/L	2.55 ± 0.97	2.54 ± 1.01	0.992
CK‐MB, ng/mL	1.80 (1.30,2.30)	1.80 (1.30,2.50)	0.478
Cr, umol/L	83.5 (69.9,93.8)	80.2 (67.6,92.9)	0.464
eGFR	78.9 ± 19.2	81.1 ± 18.8	0.397
Uric acid, umol/L	368 ± 106	353 ± 84.4	0.208
BUN, mmo/L	5.74 (4.79,6.98)	5.62 (4.81,6.92)	0.900
K, mmol/L	4.35 ± 0.41	4.26 ± 0.34	0.081
TSH, mIU/L	1.98 (1.23,2.89)	1.95 (1.31,3.07)	0.957
D‐Dimer, ng/mL	59.0 (36.5,102)	57.5 (39.0,110)	0.684

*Note*: *p*<0.05*, *p*<0.001**. Values are presented as n (%), mean ± SD or median (IQR).

Abbreviations: AAD_blank, using anti‐arrhythmic drugs in blanking period; AAD_post, using anti‐arrhythmic drugs after blanking period; AAD_pre, using anti‐arrhythmic drugs before ablation; ACEI, angiotensin‐converting enzyme inhibitors; AF, atrial fibrillation; ARB, angiotensin receptor blockers; BMI, body mass index; BNP, B‐type natriuretic peptide; BUN, blood urea nitrogen; CAD, coronary heart disease; CCB, Calcium channel blockers; CK‐MB, creatine kinase muscle and brain isoenzymes; Cr, creatinine; DBP, diastolic blood pressure; eGFR, estimated glomerular filtration rate; hsCRP, high sensitivity C‐reactive protein; HR, heart rate; LDL, low density lipoprotein; LAVI, left atrium volume index; LVEF, left ventricular ejection fraction; LVEDD, left ventricular end‐diastolic dimension; NLR, Neutrophil‐to‐lymphocyte ratio; SBP, systolic blood pressure; TNFSF14, tumor necrosis factor superfamily protein 14; TSH, thyrotropin.

Patients who experienced AF recurrence had a higher proportion of persistent AF (*p* = 0.007), a longer duration of AF (*p* = 0.020), and a higher plasma levels of high‐sensitivity C‐reactive protein (CRP) (*p* = 0.040). They also presented with larger left atrial volume index (LAVI) (*p* < 0.001) and left ventricular end‐diastolic dimension (*p* = 0.001). What's more, the use of anti‐arrhythmic drugs before ablation was significantly more common in patients with recurrence (*p* = 0.009).

**Figure 2:**
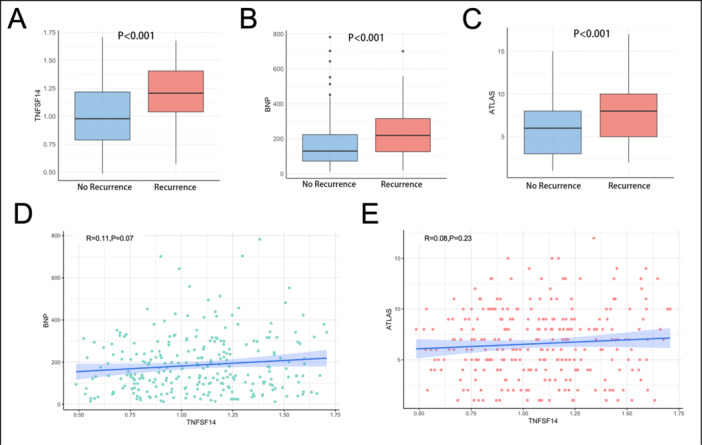
TNFSF14 was significantly elevated in patients with AF recurrence. A) Comparison of plasma TNFSF14 levels between recurrence and non‐recurrence groups. (B) Comparison of plasma BNP levels between recurrence and non‐recurrence groups. (C) Comparison of ATLAS score between recurrence and non‐recurrence groups. (D) Correlation analysis between plasma levels of TNFSF14 and BNP. (E) Correlation analysis between plasma TNFSF14 levels and ATLAS score. AF = atrial fibrillation; BNP = B‐type natriuretic peptide; TNFSF14 = tumor necrosis factor superfamily protein 14.

Moreover, plasma level of TNFSF14 (1.21 ± 0.24 vs. 1.02 ± 0.29 ng/mL, *p* < 0.001) (Figure 2A) and BNP (*p* < 0.001) (Figure 2B) were significantly higher in patients with recurrence during follow‐up. Besides, ATLAS score was significantly higher in recurrence group (*p* < 0.001) (Figure 2C). However, no significant correlation was observed between TNFSF14 levels and BNP (Pearson R = 0.11, *p* = 0.070) (Figure 2D), or ATLAS score (Pearson R = 0.08, *p* = 0.229) (Figure 2E).

### 3.2 TNFSF14 Is an Independent Risk Factor for AF Recurrence Post‐Ablation

Using the predefined cut‐off value (1.09 ng/mL; sensitivity 71.6%, specificity 59.9%), TNFSF14 was converted into a categorical variable and subjected to univariate Cox regression analysis. Univariate Cox regression analysis demonstrated that ATLAS score, AF Duration, AF classification (persistent AF vs paroxysmal AF), LAVI, left ventricular end‐diastolic dimension, use of anti‐arrhythmic drugs, and plasma levels of BNP and TNFSF14 were potential and independent predictors of AF recurrence (all *p* < 0.05, Table 2). Other variables showed no significant relationships with AF recurrence, as verified by univariate Cox analysis (Supporting Information S1: Table 1). These significant variables were then included in multivariate Cox regression analysis, which demonstrated that elevated TNFSF14 levels were independently associated with an increased risk of AF recurrence (adjusted hazard ratio [HR]: 3.65, 95% confidence interval [CI]: 2.19–6.09, *p* < 0.001). Additionally, the ATLAS score (HR: 1.11, 95% CI: 1.03–1.20, *p* = 0.010), LAVI (HR: 1.02, 95% CI: 1.00–1.04, *p* = 0.040), and BNP (HR: 1.00, 95% CI: 1.00–1.00, *p* = 0.010) were also identified as independent risk factors for AF recurrent in patients undergoing CA, as shown in Table 2.

**Table 2:** Univariate and multivariate Cox analysis.

	Univariate Cox analysis		Multivariate Cox analysis	
	HR (95% CI)	P	HR (95% CI)	P
TNFSF14	4.17 (2.53–6.87)	< 0.001	3.65 (2.19–6.09)	< 0.001
ATLAS score	1.17 (1.09–1.24)	< 0.001	1.11 (1.03–1.20)	0.010
AF Duration	1.04 (1.01–1.07)	0.008	1.03 (0.99–1.07)	0.110
AF classification	1.84 (1.18–2.87)	0.007	0.74 (0.42–1.33)	0.314
LAVI	1.04 (1.03–1.06)	< 0.001	1.02 (1.00–1.04)	0.040
LVEDD	1.06 (1.02–1.10)	0.003	1.04 (1.00–1.09)	0.086
AAD_pre	1.99 (1.21–3.27)	0.007	1.80 (1.05–3.07)	0.052
BNP	1.00 (1.00–1.00)	< 0.001	1.00 (1.00–1.00)	0.010

Abbreviations: AAD_pre, use of anti‐arrhythmic drugs before ablation; AF, atrial fibrillation; BNP, B‐type natriuretic peptide; LAVI, left atrium volume index; LVEDD, left ventricular end‐diastolic dimension; TNFSF14, tumor necrosis factor superfamily protein 14.

**Figure 3:**
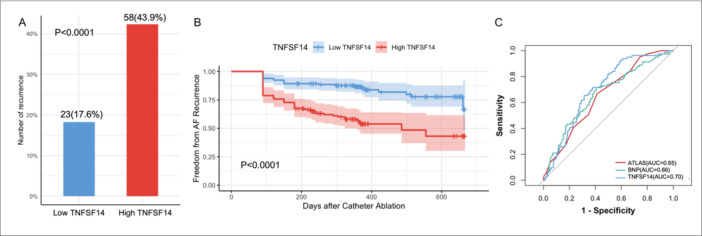
The rate of AF recurrence was higher in the high TNFSF14 group. (A) The rate of AF recurrence in patients stratified by TNFSF14 levels. (B) Kaplan‐Meier Curves for AF recurrence Stratified by TNFSF14. (C) ROC curves comparing the predictive performance of ATLAS score, BNP and TNFSF14. AF = atrial fibrillation; TNFSF14 = tumor necrosis factor superfamily protein 14.

To investigated the predictive value of TNFSF14 in AF recurrence, we conducted a comparative analysis between the two groups. In the high TNFSF14 group, the proportion of patients who underwent recurrence (59/127, 46.5%) were significantly higher than that in the low TNFSF14 group (22/136, 16.2%) (*p* < 0.001, Figure 3A). Furthermore, Kaplan‐Meier survival curves revealed significant differences in AF recurrence rates between the two TNFSF14 groups (*p* < 0.001, Figure 3B). The predictive capability of TNFSF14 was further evaluated using receiver operating characteristic (ROC) analysis (Figure 3C). The area under the ROC curve (AUC) for TNFSF14 was 0.70 (95% CI: 0.63–0.76, *p* < 0.001), compared to 0.65 (95% CI: 0.59–0.72, *p* < 0.001) for the ATLAS score, 0.66 (95% CI: 0.59–0.74, *p* < 0.001) for BNP, indicating that TNFSF14 has better predictive validity.

Sensitivity analyses yielded consistent results regardless of the grouping method (based on the mean, cut‐off of ROC, tertiles, or quartiles), confirming the robustness of the association between TNFSF14 levels and AF recurrence (Supporting Information S1: Table 2A–D, Supporting Information S1: Figure 1).

### 3.3 TNFSF14 as an Additional Biomarker for the Prediction of AF Recurrence

**Figure 4:**
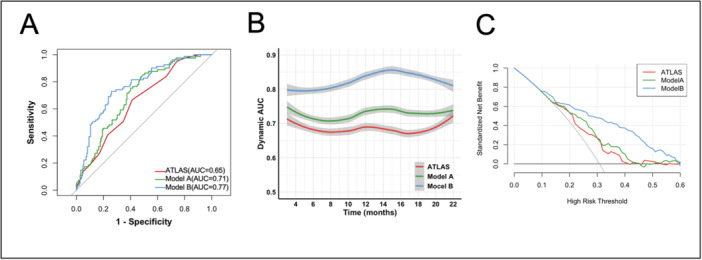
Models for predicting AF recurrence post‐ablation. (A) ROC curves comparing the predictive performance of ATLAS score, Model A and Model B. (B) Dynamic AUC analysis assessing the temporal stability of ATLAS score, Model A and Model B. (C) DCA comparing the clinical utility between ATLAS score, Model A and Model B. Model A was constructed by incorporating BNP into the ATLAS score. Model B was constructed by incorporating TNFSF14 into the Model A.

Based on the ATLAS score, two new models were constructed for predicting AF recurrence. Model A combined BNP and ATLAS score, whereas Model B integrated TNFSF14 with the Model A. The performance of these models was assessed using ROC curves, as illustrated in Figure 4A. Notably, Model B, which incorporated TNFSF14 into Model A, demonstrated the highest predictive accuracy (AUC: 0.77, 95% CI: 0.71–0.83; ΔAUC = 0.06, *p* = 0.009).

To further evaluate these Models from multiple perspectives, we conducted additional analyses. The time‐dependent AUC curve demonstrated that Model B was a stronger predictor of AF recurrence after CA than the ATLAS score and Model A, with temporal stability (Figure 4B). Decision curve analysis demonstrated that Model B provided a greater net benefit for predicting AF recurrence compared to the ATLAS score or Model A (Figure 4C). Further analysis confirmed the added value of incorporating TNFSF14 into Model A, resulting in improved predictive accuracy, with a net reclassification improvement (NRI) of 36.3% (*p* < 0.001) and an integrated discrimination improvement (IDI) of 8.10% (*p* = 0.012) (Table 3).

**Table 3:** Improvement in predictive accuracy for AF recurrence after addition of TNFSF14 and BNP to the ATLAS score.

Model	C‐statistic	P	NRI (95% CI)	P	IDI (95% CI)	P
ATLAS	0.68	< 0.001	Ref.	—	Ref.	—
Model A	0.70	< 0.001	0.18 (0.01–0.35)	0.034	0.03 (−0.01 to 0.08)	0.096
ATLAS	0.68	< 0.001	Ref.	—	Ref.	—
Model B	0.77	< 0.001	0.44 (0.19–0.57)	0.002	0.12 (0.05–0.18)	0.004
Model A	0.70	< 0.001	Ref.	—	Ref.	—
Model B	0.77	< 0.001	0.36 (0.20–0.56)	< 0.001	0.08 (0.02–0.14)	0.012

*Note*: Model A: ATLAS score + BNP. Model B: ATLAS score + BNP + TNFSF14. The colors in table are only intended to facilitate the reader's distinction between the models being compared for NRI and IDI.

Abbreviations: CI, confidence interval; IDI, integrated discrimination improvement; NRI, net reclassification improvement.

## 4 Discussion

In this study, we prospectively investigated the predictive value of TNFSF14 levels for AF recurrence after CA. To our knowledge, this is the first study to explore the relationship between elevated TNFSF14 levels and AF recurrence. As shown in this study, the TNFSF14 levels were significantly elevated in patients who experienced recurrence, and were identified as independent risk factors for the AF recurrence. Besides, TNFSF14 demonstrated superior predictive value compared to the traditional ATLAS score [10], with predictive efficacy increasing over time. Combining TNFSF14 levels with BNP levels enhanced the predictive performance of existing models like the ATLAS score in predicting recurrence. Thus, TNFSF14 is strongly associated with AF recurrence after CA, and holds promise as a biomarker for predicting the risk of postoperative recurrence.

Atrial remodeling is a key mechanism in the initiation, maintenance, and recurrence of AF [4, 11], which is associated with fibrosis and inflammation. Firstly, inflammation can initiate AF, which subsequently generates an inflammatory response that further enhances atrial remodeling and perpetuates the arrhythmia—the so‐called “AF begets AF” phenomenon [12]. TNFSF14 has been found to be associated with inflammatory factors such as IL‐6 and CRP, which are involved in the inflammatory cascade response in patients with atrial fibrillation [13].

Furthermore, fibrosis is a crucial pathophysiological mechanism of atrial remodeling and significantly contributes to the recurrence of atrial fibrillation [14]. TNFSF14 promotes structural cell proliferation and the expression of chemokines, growth factors, and metalloproteinases by binding to herpesvirus entry mediator (HVEM) or lymphotoxin β receptor (LTβR) [15, 16]. In addition, TNFSF14's interaction with HVEM and LTβR on inflammatory cells stimulates the secretion of pro‐fibrotic factors such as TGFβ1, IL‐13, and TSLP, indirectly promoting atrial fibrosis [17, 18, 19, 20]. The latter is achieved by the activation of genes associated with inflammatory via classical and non‐canonical NFκB pathways [21, 22, 23].

However, the above mechanisms have mostly been studied in non‐myocardial tissues or organs (e.g., bone, lungs, livers, skin, airways), and whether they are suitable for atrial remodeling needs to be further explored in the future.

Previous research has demonstrated that TNFSF14 serves as a prognostic marker in cardiovascular diseases, particularly atherogenesis [24, 25, 26]. Furthermore, TNFSF14 is overexpressed in coronary artery disease patients, potentially due to its pro‐inflammatory and pro‐lipogenesis functions via NF‐κB pathway activation [27, 28]. Additionally, TNFSF14 may sever as a biomarker for stable coronary artery disease prognosis [29]. TNFSF14 is also implicated in chronic heart failure progression [30]. In the context of AF, TNFSF14 is highly expressed in AF patients, which may be due to its promotion of cardiac fibrosis and vulnerability of AF via PI3Kγ/SGK1 pathway‐dependent M2 macrophage polarization [9]. This study is the first to establish TNFSF14 as an independent biomarker for predicting AF recurrence. It is found that TNFSF14 is an independent biomarker for predicting the recurrence of AF.

Existing models for predicting AF recurrence primarily incorporate clinical information, with limited consideration of biomarker. In this study, we innovatively integrated TNFSF14 and BNP—two biomarkers identified as significant in multivariate Cox regression analysis—into the established ATLAS score. The results demonstrated that the newly constructed model significantly improved the predictive performance of previous models, potentially because these biomarkers capture key pathophysiological mechanisms of AF recurrence, such as fibrosis and inflammation, which were not accounted for in earlier models. However, despite these findings, the predictive potential of this model requires further validation in future studies to confirm its applicability and enhance its applicability and enhance its robustness across different populations.

### 4.1 Study Limitations

Our study has several limitations. Firstly, it is a single‐center study with a relatively small sample size, so the results require validation in large, multi‐center prospective trials. Nevertheless, it is the first study to discover the relationship between TNFSF14 and AF recurrence, providing new insights for the management of AF. Secondly, although TNFSF14 was significantly associated with AF recurrence, its predictive ability was relatively limited (sensitivity 71.6%, specificity 59.9%), which may restrict its clinical applicability as a standalone biomarker. This finding may partly result from the complex mechanisms underlying AF recurrence; nevertheless, as demonstrated in this study, TNFSF14 may still provide additional predictive value when incorporated into multivariable models. Moreover, this study was unable to use implantable cardiac monitoring to assess AF recurrence, which may have led to the omission of asymptomatic AF episodes, potentially leading to an overestimation of ablation success. However, evidence suggests that intermittent monitoring, when accumulated over time, can provide estimates of AF burden comparable with ICM [31]. Therefore, this study conducted close follow‐up with regular clinical visits, ECG and 24‐h Holter ECG monitoring to closely approximate the true recurrence rate. Fourthly, plasma levels of TNFSF14 were measured only at baseline, postoperative levels or temporal changes during recurrence were not assessed. Additionally, we did not perform intraoperative mapping of fibrosis, which could offer a direct correlation between TNFSF14 levels, the extent of fibrosis, and AF recurrence. These will be further investigated in future studies.

## 5 Conclusion

In this analysis of patients with AF, baseline TNFSF14 levels are a strong predictor of AF recurrence following CA. Incorporating TNFSF14 into existing predictive models improves their accuracy, highlighting its potential use in personalized patient management for AF.

## Conflicts of Interest

The authors declare that the research was conducted in the absence of any commercial or financial relationships that could be construed as a potential conflict of interest.

## Supporting information


**Figure S1:** The Rate of AF Recurrence Differed Significantly Among Groups Stratified by TNFSF14 Levels. **Table S1:** Univariate Cox analysis(P ≥ 0.05). **Table S2A:** Univariate and Multivariate Cox analysis (Grouping by the Mean). **Table S2B:** Univariate and Multivariate Cox analysis (Grouping by the cut‐off of ROC). **Table S2C:** Univariate and Multivariate Cox analysis (Grouping by Tertiles). **Table S2D:** Univariate and Multivariate Cox analysis (Grouping by Quartiles).

## Data Availability

The authors have nothing to report.
