# Supplementary material for: Tumor Necrosis Factor Superfamily 14: A Potential Predictor of Atrial Fibrillation Recurrence After Catheter Ablation
Source: Clin Cardiol. 2026 Apr 20;49(4):e70283. doi: 10.1002/clc.70283 (PMC13094365; doi:10.1002/clc.70283)
Supplement: Supplementary file 1 — Figure S1: The Rate of AF Recurrence Differed Significantly Among Groups Stratified by TNFSF14 Levels. Table S1: Univariate Cox analysis(P ≥ 0.05). Table S2A: Univariate and Multivariate Cox analysis (Grouping by the Mean). Table S2B: Univariate and Multivariate Cox analysis (Grouping by the cut‐off of ROC). Table S2C: Univariate and Multivariate Cox analysis (Grouping by Tertiles). Table S2D: Univariate and Multivariate Cox analysis (Grouping by Quartiles). [file CLC-49-e70283-s001.docx]

**Supplementary material**

**Tumor Necrosis Factor Superfamily 14: A Potential Predictor of Atrial Fibrillation Recurrence After Catheter Ablation**

**Dongtao Zhou, MD, Tong Liu, MD, Fang Liu, MD, Mengmeng Li, MD, Chenxi Jiang, MD, Ribo Tang, MD, Wei Wang, MD, Xin Zhao, MD, Changyi Li, MD, Changqi Jia, MD, Man Ning, MD, Li Feng, MD, Dan Wen, MD, Jing Lin, MD, Hui Zhu, MD, Yuexin Jiang, MD, Xueyuan Guo, MD, Songnan Li, MD, Chao Jiang, MD, Ning Zhou, MD, Caihua Sang, MD, Deyong Long, MD^*^, Xin Du, MD, Jianzeng Dong, MD, Changsheng Ma, MD**

**Contents**

[Samples and analyses 2](#_Toc475443674)

[ATLAS score 3](#_Toc2142662078)

[Table S1 4](#_Toc568268403)

[Table S2 5](#_Toc1027271012)

[Figure S1 6](#_Toc1722860451)

# **Supplemental Methods**

# **Samples and analyses**

All venous blood samples were collected at baseline (prior to ablation). Blood was drawn from the antecubital vein into EDTA tubes and centrifuged at 3000 g for 15 minutes within 2 hours after collection. The supernatant plasma was aliquoted, frozen at −20°C, and then transferred to –80°C for long-term storage within one week until analysis.

Tumor Necrosis Factor Superfamily 14 (TNFSF14) was quantified by enzyme-linked immunosorbent assay (ELISA) using a commercially available ELISA kit (Human TNFSF14 ELISA Kit, YX-201406H, Cloud-Clone Corp, China), following the manufacturer's instructions. The kit employs a one-step double-antibody sandwich method. After the addition of the TMB substrate and stop solution, the optical density (OD) was measured at 450 nm using a microplate reader.Standard curves were generated using serial dilutions of recombinant human TNFSF14, and plasma concentrations were determined by extrapolation using a log–log linear regression model.

The kit performance was validated as follows: accuracy, with the correlation coefficient (R) between the standard curve and expected concentrations ≥0.9900; sensitivity, with a minimum detectable concentration <15 pg/mL; specificity, showing no cross-reactivity with other soluble structural analogs; and reproducibility, with intra- and inter-assay coefficients of variation (CV) both <15%.

# **ATLAS score**

The ATLAS score^1^ is a scoring system developed to predict the risk of atrial fibrillation (AF) recurrence after the first catheter ablation, derived from a registry of 1,934 patients from two centers. The score demonstrated good predictive performance, with a c-statistic of 0.75 in both the development and validation cohorts. Our study further validated its predictive ability for AF recurrence while extending its application to patients undergoing non-first-time ablation, thereby broadening its scope of use. The variables included in the score are as follows:

| Variable | Number of points |
| --- | --- |
| **A**ge > 60 years | 1 |
| **T**ype of AF (non-paroxysmal) | 2 |
| **L**eft **a**trial volume indexed to BSA | 1 point per each 10 mL/m^2^(rounded to nearest integer) |
| **S**ex (female) | 4 |
| **S**moking (current smoking) | 7 |

BSA=Body Surface Area

Example: A 54 years-old non-smoker woman with persistent AF and an LA volume of 57 mL/m^2^ would have an ATLAS score of 12(=0+2+6+4+0).

1. Mesquita J, Ferreira AM, Cavaco D, et al. Development and validation of a risk score for predicting atrial fibrillation recurrence after a first catheter ablation procedure - ATLAS score. Europace. 2018;20(FI_3):f428-f435.

**Table S1. Univariate Cox analysis(P** ≥ **0.05)**

|  | HR(95%CI) | P |
| --- | --- | --- |
| Age | 1.00(0.98-1.02) | 0.974 |
| Sex | 0.90(0.58-1.40) | 0.644 |
| Height | 1.02(1.00-1.05) | 0.111 |
| Weight | 1.00(0.99-1.02) | 0.833 |
| BMI | 1.00(0.94-1.07) | 0.912 |
| HR | 0.99(0.97-1.01) | 0.273 |
| SBP | 1.00(0.99-1.01) | 0.888 |
| DBP | 1.00(0.98-1.01) | 0.630 |
| Redo | 1.34(0.99-1.80) | 0.056 |
| CHA2DS2-VASC | 1.04(0.91-1.19) | 0.538 |
| HAS-BLED | 1.25(0.95-1.66) | 0.117 |
| Current smoking | 0.97(0.50-1.89) | 0.933 |
| Current alcohol drinking | 0.73(0.43-1.23) | 0.231 |
| Heart failure | 0.79(0.48-1.30) | 0.360 |
| Ischemic stroke | 0.68(0.36-1.29) | 0.238 |
| Hypertension | 1.04(0.67-1.60) | 0.871 |
| CAD | 1.15(0.67-1.99) | 0.612 |
| Diabetes | 0.98(0.55-1.74) | 0.937 |
| Hyperlipidemia | 0.80(0.50-1.28) | 0.352 |
| Thyroid gland disease | 0.56(0.27-1.17) | 0.124 |
| LVEF | 0.98(0.96-1.01) | 0.133 |
| ACEI/ARB | 1.47(0.94-2.28) | 0.090 |
| β blocker | 0.82(0.50-1.33) | 0.413 |
| CCB | 1.14(0.66-1.97) | 0.629 |
| hsCRP | 0.99(0.96-1.03) | 0.586 |
| NLR | 1.08(0.90-1.30) | 0.399 |
| LDL | 0.95(0.76-1.17) | 0.608 |
| CK-MB | 0.90(0.70-1.15) | 0.399 |
| Cr  eGFR | 1.00(1.00-1.00)  1.00(0.98-1.01) | 0.853  0.406 |
| Uric acid | 1.00(1.00-1.00) | 0.246 |
| BUN | 1.00(0.90-1.11) | 0.997 |
| K | 1.69(0.93-3.07) | 0.087 |
| TSH | 1.04(0.94-1.14) | 0.479 |
| D-Dimer | 1.00(1.00-1.00) | 0.427 |

BMI = body mass index; HR = heart rate; SBP = systolic blood pressure; DBP = diastolic blood pressure; CAD = coronary heart disease; LVEF = left ventricular ejection fraction; CCB = Calcium channel blockers; ACEI = angiotensin-converting enzyme inhibitors; ARB = angiotensin receptor blockers; hsCRP = high sensitivity C-reactive protein; NLR = Neutrophil-to-lymphocyte ratio; LDL = low density lipoprotein; CK-MB = creatine kinase muscle and brain isoenzymes; Cr = creatinine; eGFR = estimated glomerular filtration rate; BUN = blood urea nitrogen; TSH = thyrotropin.

**Table S2A. Univariate and Multivariate Cox analysis (Grouping by the Mean)**

Univariate Cox analysis Multivariate Cox analysis

|  | HR(95%CI) | P | HR(95%CI) | P |
| --- | --- | --- | --- | --- |
| TNFSF14 | 3.17(1.94-5.17) | <0.001 | 2.89 (1.75-4.76) | <0.001 |
| ATLAS score | 1.17(1.09-1.24) | <0.001 | 1.11 (1.03-1.20) | 0.009 |
| AF Duration | 1.04 (1.01-1.07) | 0.008 | 1.03 (1.00-1.07) | 0.091 |
| AF classification | 1.84 (1.18-2.87) | 0.007 | 0.68 (0.38-1.21) | 0.186 |
| LAVI | 1.04 (1.03-1.06) | <0.001 | 1.02 (1.00-1.04) | 0.024 |
| LVEDD | 1.06 (1.02-1.10) | 0.003 | 1.04 (1.00-1.09) | 0.051 |
| AAD | 1.99 (1.21-3.27) | 0.007 | 1.79 (1.04-3.05) | 0.034 |
| BNP | 1.00 (1.00-1.00) | <0.001 | 1.00 (1.00-1.00) | 0.002 |

**Table S2B. Univariate and Multivariate Cox analysis (Grouping by the cut-off of ROC)**

Univariate Cox analysis Multivariate Cox analysis

|  | HR(95%CI) | P | HR(95%CI) | P |
| --- | --- | --- | --- | --- |
| TNFSF14 | 3.88(2.43-6.19) | <0.001 | 3.17 (1.96-5.14) | <0.001 |
| ATLAS score | 1.17(1.09-1.24) | <0.001 | 1.11 (1.02-1.20) | 0.012 |
| AF Duration | 1.04 (1.01-1.07) | 0.008 | 1.03 (1.00-1.07) | 0.094 |
| AF classification | 1.84 (1.18-2.87) | 0.007 | 0.71 (0.40-1.27) | 0.245 |
| LAVI | 1.04 (1.03-1.06) | <0.001 | 1.02 (1.00-1.04) | 0.048 |
| LVEDD | 1.06 (1.02-1.10) | 0.003 | 1.05 (1.00-1.09) | 0.047 |
| AAD | 1.99 (1.21-3.27) | 0.007 | 1.65 (0.97-2.79) | 0.065 |
| BNP | 1.00 (1.00-1.00) | <0.001 | 1.00 (1.00-1.00) | 0.007 |

**Table S2C. Univariate and Multivariate Cox analysis (Grouping by Tertiles)**

Univariate Cox analysis Multivariate Cox analysis

|  | HR(95%CI) | P | HR(95%CI) | P |
| --- | --- | --- | --- | --- |
| TNFSF14 | 2.40(1.76-3.25) | <0.001 | 2.21 (1.62-3.02) | <0.001 |
| ATLAS score | 1.17(1.09-1.24) | <0.001 | 1.12 (1.03-1.21) | 0.006 |
| AF Duration | 1.04 (1.01-1.07) | 0.008 | 1.04 (1.00-1.08) | 0.060 |
| AF classification | 1.84 (1.18-2.87) | 0.007 | 0.73 (0.41-1.30) | 0.286 |
| LAVI | 1.04 (1.03-1.06) | <0.001 | 1.02 (1.00-1.03) | 0.095 |
| LVEDD | 1.06 (1.02-1.10) | 0.003 | 1.04 (1.00-1.09) | 0.057 |
| AAD | 1.99 (1.21-3.27) | 0.007 | 1.86 (1.09-3.16) | 0.023 |
| BNP | 1.00 (1.00-1.00) | <0.001 | 1.00 (1.00-1.00) | 0.005 |

**Table S2D. Univariate and Multivariate Cox analysis (Grouping by Quartiles)**

Univariate Cox analysis Multivariate Cox analysis

|  | HR(95%CI) | P | HR(95%CI) | P |
| --- | --- | --- | --- | --- |
| TNFSF14 | 1.92(1.54-2.40) | <0.001 | 1.79 (1.43-2.25) | <0.001 |
| ATLAS score | 1.17(1.09-1.24) | <0.001 | 1.11 (1.03-1.21) | 0.008 |
| AF Duration | 1.04 (1.01-1.07) | 0.008 | 1.04 (1.00-1.08) | 0.053 |
| AF classification | 1.84 (1.18-2.87) | 0.007 | 0.71 (0.40-1.27) | 0.250 |
| LAVI | 1.04 (1.03-1.06) | <0.001 | 1.01 (1.00-1.03) | 0.126 |
| LVEDD | 1.06 (1.02-1.10) | 0.003 | 1.04 (1.00-1.09) | 0.081 |
| AAD | 1.99 (1.21-3.27) | 0.007 | 1.94 (1.14-3.32) | 0.015 |
| BNP | 1.00 (1.00-1.00) | <0.001 | 1.00 (1.00-1.00) | 0.005 |

TNFSF14 = tumor necrosis factor superfamily protein 14; AF = atrial fibrillation; LAVI = left atrium volume index; LVEDD = left ventricular end-diastolic dimension; AAD = anti-arrhythmic drugs; BNP = B-type natriuretic peptide；

**Figure S1. The Rate of AF Recurrence Differed Significantly Among Groups Stratified by TNFSF14 Levels.**


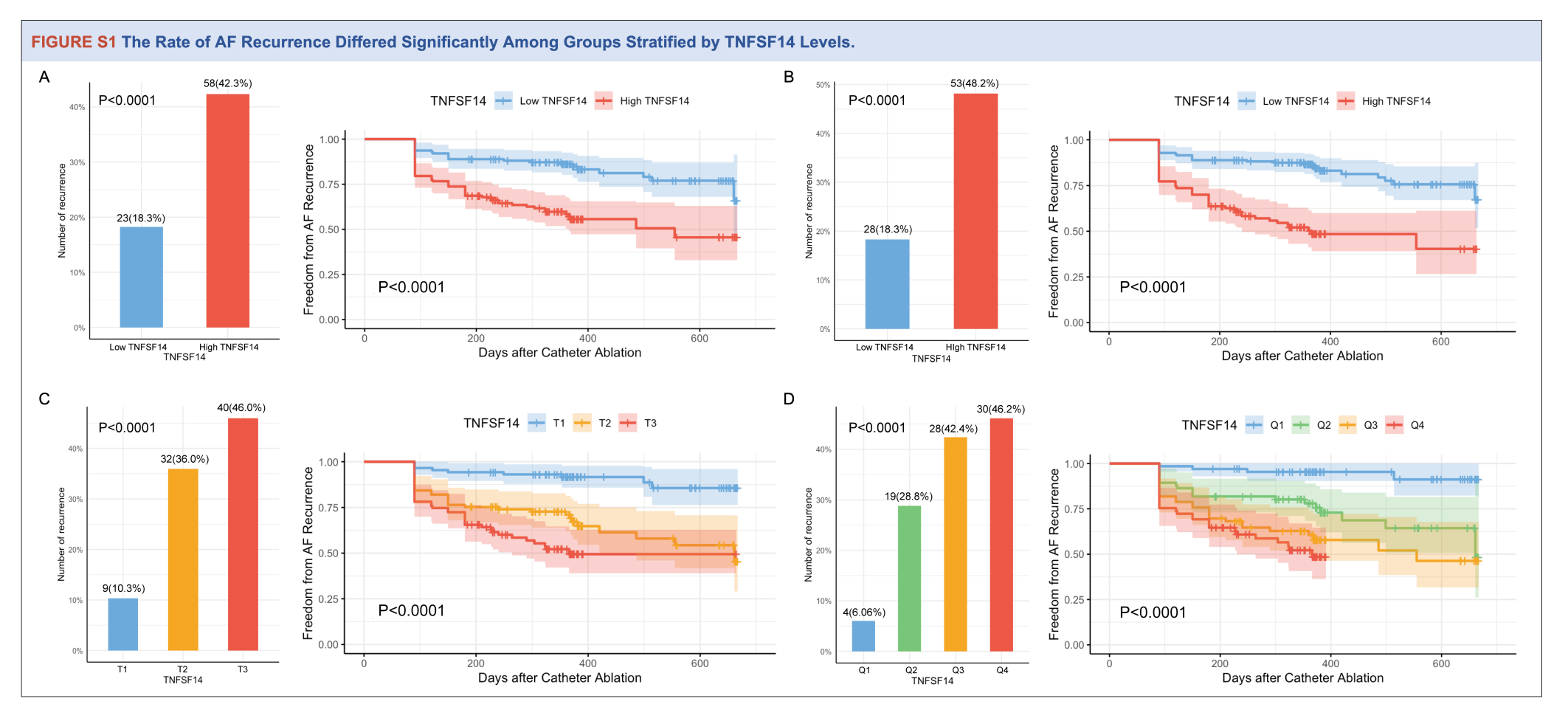


A–D show atrial fibrillation (AF) recurrence in patients stratified by TNFSF14 levels using different grouping methods: (A) by the mean, (B) by the cut-off of ROC, (C) by tertiles, and (D) by quartiles. The left panels illustrate recurrence rates using bar charts, while the right panels display Kaplan–Meier curves for AF recurrence between groups in each stratification.
